# Nur77 exacerbates PC12 cellular injury *in vitro* by aggravating mitochondrial impairment and endoplasmic reticulum stress

**DOI:** 10.1038/srep34403

**Published:** 2016-09-29

**Authors:** Huimin Gao, Zhaoyu Chen, Yongmei Fu, Xiaoyan Yang, Ruihui Weng, Rui Wang, Jianjun Lu, Mengqiu Pan, Kunlin Jin, Chris McElroy, Beisha Tang, Ying Xia, Qing Wang

**Affiliations:** 1 Department of Neurology, The Third Affiliated Hospital of Sun Yat-Sen University, Tianhe Road 600, Guangzhou, Guangdong 510630, P.R. China; 2 Department of Emergency, The Third Affiliated Hospital of Sun Yat-Sen University, Tianhe Road 600, Guangzhou, Guangdong 510630, P.R. China; 3 Department of Neurology, Guangdong 999 Brain Hospital, Guangzhou 510510, China; 4 Department of Pharmacology and Neuroscience, University of North Texas Health Science Center, Fort Worth, TX 76107, USA; 5 The State Key Laboratory of Medical Genetics, Central South University, Changsha, Hunan 410078, China; 6 Department of Neurosurgery, The University of Texas McGovern Medical School, TX 77030, USA; 7 Guangdong Province Key Laboratory of Brain Function and Disease, Guangzhou, Guangdong, P.R. China

## Abstract

The nuclear orphan receptor, Nur77 plays important roles in neuroimflammation, apoptosis, and dopaminergic neurodegeneration. We conducted a further mechanistic investigation into the association of Nur77 with cell death. Cytosporone B (Csn-B), an agonist for Nur77, and Nur77 knockdown were adopted in the 6-hydroxydopamine (OHDA)-lesioned PC12 cells to investigate the mechanisms underlying Nur77-mediated injury. The 6-OHDA incubation caused Nur77 translocation from the nucleus to cytosol and Endoplasm reticulum (ER) and induced co-localization of Tom20/Nur77 and Protein Disulfide Isomerase (PDI)/Nur77. Nur77 activation further decreased cell viability, aggravated intracellular LDH release, intracellular Ca^2+^, ROS levels, apoptosis, ER tress and, mitochondrial transmembrane potential (ΔΨm) decline. In addition, Nur77 activation significantly enhanced the efficiency of autophagy as indicated by an up-regulation of Beclin-1/LC-3 and downregulation of p62, and aggravated mitochondrial dysfunctions and ER stress as shown by increased HSP60/Cytochrome C (Cyt C) and CHOP-ATF3 levels respectively. These changes could be partially reversed by Nur77 knockdown. Moreover, Nur77 activation upregulated PINK1 and downregulated Parkin levels. We conclude that Nur77 exacerbates PC12 cell death at least partially by aggravating the mitochondrial impairment and ER stress and enhancing autophagy. We propose that Nur77 is likely a critical target in the PD therapy.

Parkinson’s disease (PD) is mainly characterized by the dopaminergic (DA) neurodegeneration. Although the etiology of PD is far from clear, the pathogenesis of PD is generally recognized to be associated with oxidative stress, chronic neuroinflammatory responses, excitotoxicity, autophagy and apoptosis^1^,^2^,^3^,^4^,^5^. Recently, increasing evidence suggests that mitochondrial dysfunctions are directly involved in PD neuro-pathogenesis^2^,^3^,^6^. These mitochondrial dysfunctions are mainly characterized by the generation of ROS, a decrease of mitochondrial complex I activity, autophagic induction, cytochrome-c release, ATP depletion and caspase 3 activation.

Nur77, a transcription factor of the nuclear receptor, plays important roles in neuroinflammation, cell proliferation, differentiation, apoptosis, metabolism and development^2^,^7^,^8^,^9^,^10^. In humans, three family members have been identified: nerve growth factor IB (also known as Nur77, NR4A1, or TR3), Nuclear receptor related 1 (also known as Nurr1, or NR4A2), and neuron-derived orphan receptor 1 (Nor-1, NR4A3). Among the three members, Nurr1 was firstly discovered to be associated with familial parkinsonism, and functions mainly in transcriptional activation to regulate a battery of genes expressed in dopaminergic (DA) neurons^11^; On the other hand, Nur77 is less investigated in terms of its role in PD. Nur77 was initially classified as an immediate-early response gene as it can be rapidly induced by diverse signals, including growth factors, cytokines, peptide hormones, neurotransmitters and stress^12^. Our previous and other studies have demonstrated that in the *in vitro* PD model, Nur77 translocation from the nucleus to cytosol induced co-localization of Cyt C/HSP60/Nur77 in the cytosol and modulated mitochondrial impairment, subsequently leading to the neuronal death^2^,^13^. Several lines of recent evidence have indicated the possible associations among Nur77, mitochondrial dysfunctions and autophagic cellular death in cancer cells^14^,^15^. However, this evidence is needed to mechanistically elucidate if Nur77 influences the neuronal survival and how it participants in the neuro-pathogenesis of PD via interacting autophagy and mitochondrial function.

Increasing evidence shows that mitochondrial dysfunction, endoplasmic reticulum stress (ERS), autophagy, and apoptosis have been critically involved in the pathogenesis of neurodegenerative disorders like PD and AD^4^,^6^,^16^,^17^,^18^,^19^. Given the roles of Nur77 in the neuroinflammation, autophagy, and mitochondrial dysfunctions in PD, we sought to mechanistically investigate: (1) the direct involvement of Nur77 in mitochondrial dysfunctions; (2) the associations between Nur77 and ER stress, and 3) the autophagy association between Nur77 verse PINK1 and Parkin in 6-OHDA-lesioned pheochromocytoma (PC12) cells.

## Results

### Nur77 exacerbated PC12 cell injury

**Figure 1:**
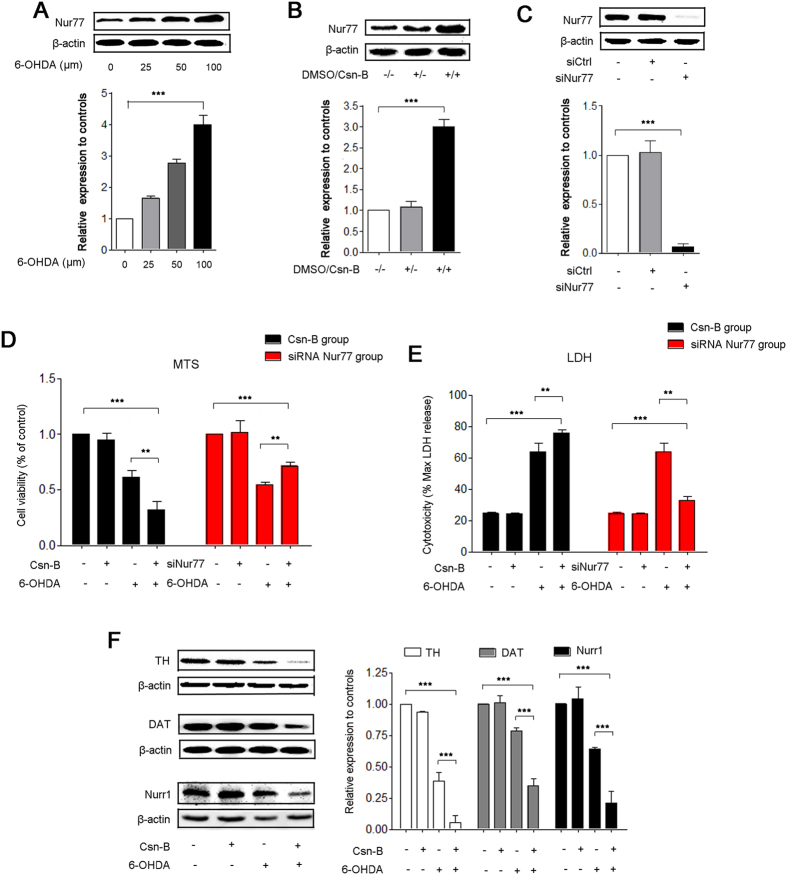
Nur77 exacerbated PC12 cell injury in 6-OHDA-lesioned model. (**A–C**) Western blots analysis shows 6 -OHDA induces a concentration- dependent (0, 25, 50, 100 μM) increase in the expression of Nur77 in PC12 cells (**A**). Under treatment with 5 μg/ml Csn-B for 24 huors, Nur77 expression increases clearly (**B**). Transfection with siRNA targeting Nur77, Nur77 decreases sharply (**C**). The bar chart shows the relative quantification of Nur77 compared with that of β-actin (**A–C**). The data are expressed as the relative ratios of the control group, which was set to 1.0, and are expressed as the mean ± SEM of 5 independent experiments, ***p < 0.001. (**D,E**) Cell viability was measured using MTS assay (**D**). LDH leakage into the medium was measured using LDH assays (**E**). For agitated Nur77, PC12 cells were incubated with 100 μM 6-OHDA or 5 μg/ml Csn-B alone, or in combination with both 6-OHDA and Csn-B, and control vehicle was used as control. For knockdown of Nur77, PC12 cells were transfected by empty vector or siRNA targeting Nur77 with or without incubation with 100 μM 6-OHDA. The MTS data are expressed as the relative ratios of the control group, which was set to 1.0. The LDH data are expressed as the ratio of LDH release level to the maximal LDH release. Data are expressed as the mean ± SEM of 5 independent experiments, **p < 0.01, ***p < 0.001. (**F**) Western blots of TH, DAT, Nurr1 expressions in PC12 after treatment with 100 μM 6-OHDA or 5 μg/ml Csn-B alone, or in combination with both 6-OHDA and Csn-B, and control vehicle was used as control. The bar chart shows the relative quantification of proteins. The data are expressed as the mean ± SEM of 5 independent experiments, **p < 0.01, ***p < 0.001.

6-OHDA significantly increased the expression of Nur77 in a dose-dependent fashion. In the group of 100 μM 6-OHDA for 24 h incubation, Nur77 increased by 4.3-fold compared to the control level (***p < 0.001, n = 5 independent experiments, Fig. 1A). Since the incubation at 100 μM of 6-OHDA for 24 hrs induces valid PD-like changes in *in vitro* PD model as shown in our previous studies^2^, we use this treatment in the subsequent experiments.

We employed Csn-B, an agonist of Nur77, which specifically binds to the ligand-binding domain of Nur77 and stimulates Nur77-dependent transactivation activity forward^20^. We also carried out an *in vitro* knockdown experiment by introducing small interfering RNA (siRNA) against Nur77 in the PC12 cells. The results showed that in the present of Csn-B, Nur77 increased by 3 fold (***p < 0.001, n = 5 experiments, Fig. 1B) as compared to the blank groups and siRNA targeting negative control (siCtrl group), while transfection of siRNA targeting Nur77 (siNur77 group) effectively decreased Nur77 to 2.6% of the control level (***p < 0.001, n = 5 experiments, Fig. 1C).

The MTS value in the group of 24-hr 6-OHDA incubation was significantly reduced to 61.4% of the control group, while Csn-B significantly exacerbated this reduction to 31.9% of the control group (***p < 0.001, n = 5 independent experiments, Fig. 1D). In contrast, Nur77 knockdown significantly attenuated this reduction (Fig. 1D). We also measured LDH in terms of cell injury in 6-OHDA-treated PC12 cells. Our results showed that Csn-B aggravated cell injury, while Nur77 knockdown attenuated the injury in 6-OHDA-treated PC12 cells (***p < 0.001, n = 5 independent experiments, Fig. 1E). Consistently, Csn-B largely exacerbated the 6-OHDA-induced decrease in the expression of TH/DAT/Nurr1(TH: 38.8% of the control in the 6-OHDA-treated group, 5.6% in the Csn-B plus 6-OHDA-treated group, n = 5 independent experiments, ***p < 0.001; DAT: 78.5% of the control in the 6-OHDA-treated group, 40.7% in the Csn-B plus 6-OHDA-treated group, n = 5 independent experiments,***p < 0.001; Nurr1: 64.3% of the control in the 6-OHDA-treated group, 20.8% in the Csn-B plus 6-OHDA-treated group, n = 5 independent experiments,***p < 0.001, Fig. 1F). Taken together, these results strongly indicated that Nur77 exacerbated neuronal cellular death via inhibiting cell proliferation and aggravated cellular injury.

### Nur77 induced cell apoptosis through Bcl2/Cytochrome C/cleaved caspase-3 pathway

**Figure 2:**
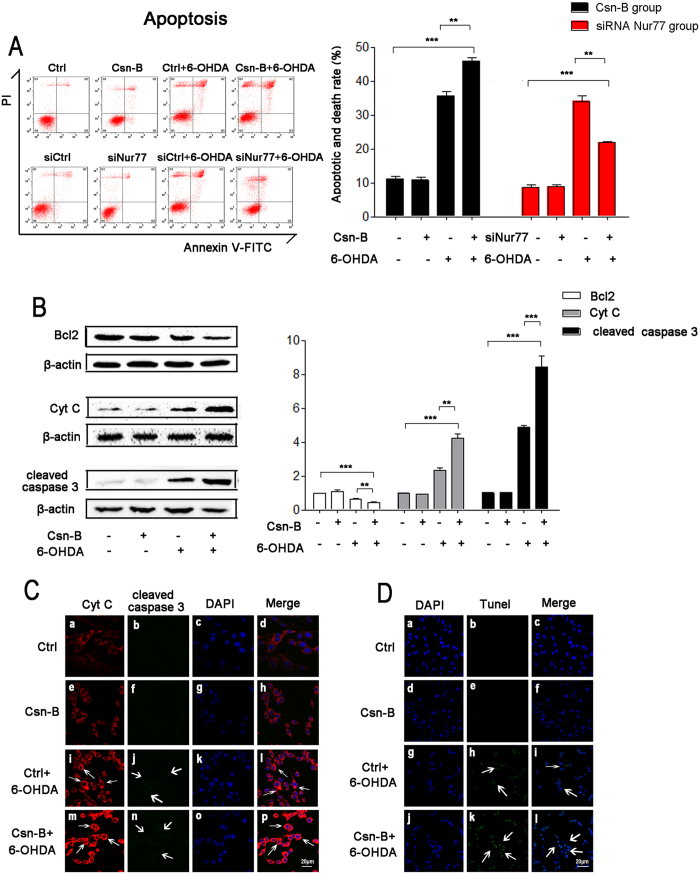
Nur77 induced cell apoptosis through Bcl2/Cytochrome C/Cleaved Caspase-3 pathway. (**A**) Apoptosis was quantified by flow cytometry with double staining annexin V-FITC and PI. For agitated Nur77, PC12 cells were incubated with 100 μM 6-OHDA or 5 μg/ml Csn-B alone, or in combination with both 6-OHDA and Csn-B, and control vehicle was used as control. For knockdown Nur77, PC12 cells were transfected by empty vector or siRNA targeting Nur77 with or without incubation with 100 μM 6-OHDA. The bar chart shows the apoptotic rate of PC12 cells. The results are expressed as the mean ± SEM of 5 independent experiments, **p < 0.01, ***p < 0.001. (**B**) Western blots of Bcl2, Cytochrome C (Cyt C), cleaved caspase 3 expressions in PC12 after treatment with 100 μM 6-OHDA or 5 μg/ml Csn-B alone, or in combination with both 6-OHDA and Csn-B, and control vehicle was used as control. The bar chart shows the relative quantification of the protein levels compared with that of β-actin. The data are expressed as the relative ratios of the blank group, which was set to 1.0, and are expressed as the mean ± SEM of 5 independent experiments. **p < 0.01, ***p < 0.001. (**C**) Immunofluorescent staining with confocal microscopy of Cyt C (red fluorescence) and cleaved caspase 3 (green fluorescence) and DAPI (blue fluorescence) after treatment with 100 μM 6-OHDA or 5 μg/ml Csn-B alone, or in combination with both 6-OHDA and Csn-B, and control vehicle was used as control. Scale bar: 20 μm. (**D**) Immunofluorescent staining with confocal microscopy of Tunel (green fluorescence) and DAPI (blue fluorescence) after treatment with 100 μM 6-OHDA or 5 μg/ml Csn-B alone, or in combination with both 6-OHDA and Csn-B, and control vehicle was used as control. Scale bar: 20 μm.

To confirm Nur77 induced cell death through apoptosis pathway, we detected apoptosis in Csn-B treated group and compared the results to those of Nur77 knockdown group using flow cytometry. Csn-B significantly exacerbated the apoptosis rate of 6-OHDA-lesioned PC12 cells (33.5% of the control group in the 6-OHDA-treated group, 47.7% in the group of 6-OHDA plus Csn-B, n = 5 independent experiments, ***p < 0.001, Fig. 2A), while Nur77 knockdown significantly attenuated the apoptosis rate (36.23% in siCtrl plus 6-OHDA-treated group and 22.3% in siNur77 plus 6-OHDA-treated group, n = 5 independent experiments,**p < 0.01, ***p < 0.001, Fig. 2A).

Western blot results showed that 6-OHDA decreased Bcl2 expression, but increased Cytochrome C and cleaved-caspase 3, while co-treatment with Csn-B amplified the alterations in the expression of these proteins (Bcl2: 65.2% of the control group in 6-OHDA-treated group, 41% in the Csn-B + 6-OHDA group; Cytochrome C: 2.3 fold of the control group in 6-OHDA-treated group, 4.2 fold in the Csn-B + 6-OHDA group, n = 5 independent experiments,***p < 0.001; cleaved-caspase 3: 4.9 fold of the control group in 6-OHDA-treated group, 8.4 fold in the Csn-B + 6-OHDA group, n = 5 independent experiments, **p < 0.01, ***p < 0.001, Fig. 2B). Similarly, the densities of Cytochrome C and cleaved-caspase 3 increased and the examination of confocal microscopy showed that they are co-located in cytoplasm (Fig. 2C). Furthermore, we performed terminal deoxynucleotidyl transferase-mediated dUTP nick end-labeling (TUNEL) assay. The TUNEL staining slightly increased in the 6-OHDA-treated group as compared to the blank group or Csn-B alone group. However, TUNEL staining was stronger in the group of Csn-B plus 6-OHDA compared to the 6-OHDA group (Fig. 2D).

### Nur77 regulated ER Stress via intracellular ROS and Ca^2+^ PI3K/AKT, CHOP and ATF3

**Figure 3:**
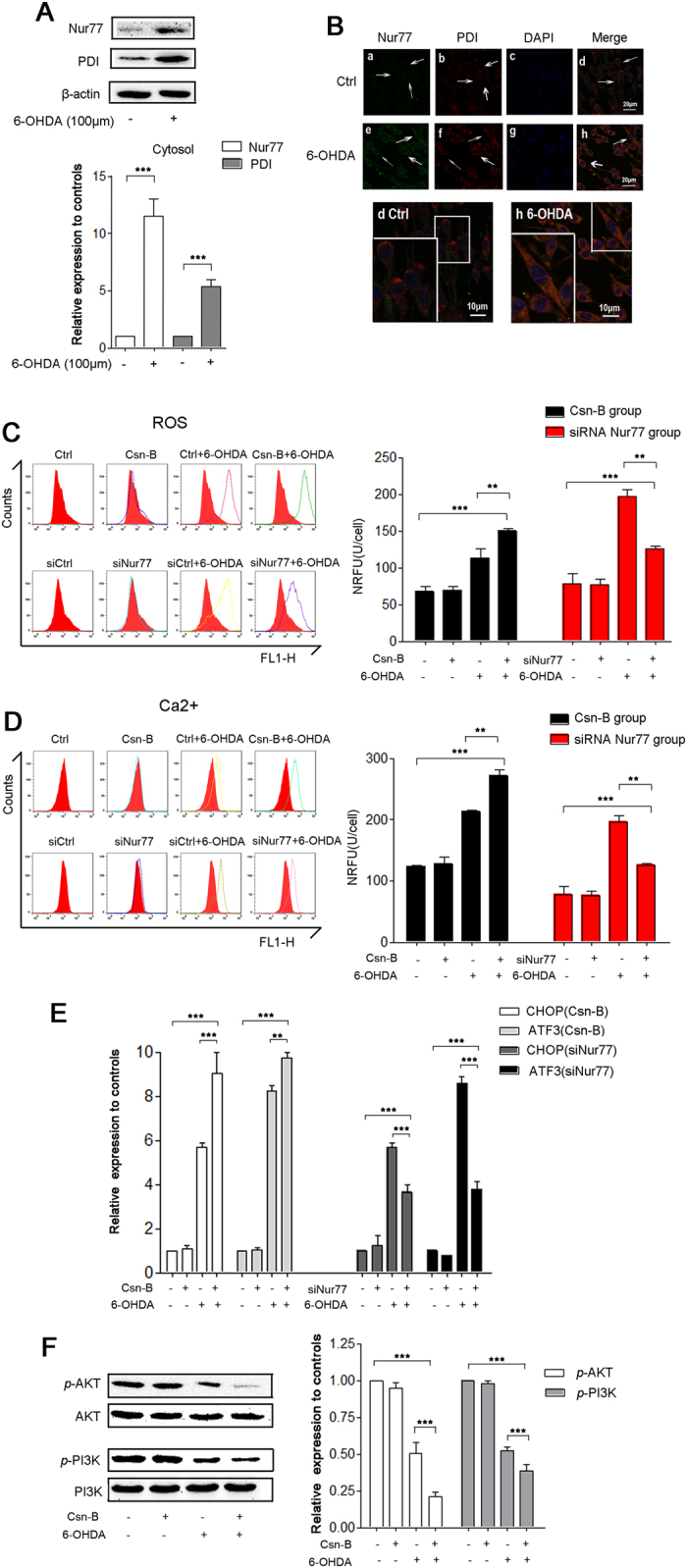
Nur77 regulated oxidative stress by increasing levels of ROS, Ca^2+^, CHOP and ATF3 mRNA. (**A**) Western blots of Nur77 and PDI in ER after treatment with 100 μM 6-OHDA for 24 hours. The bar chart shows the relative quantification of Nur77 and PDI. (**B**) Immunofluorescent staining with confocal microscopy of Nur77 (green fluorescence) and PDI (red fluorescence) and DAPI (blue fluorescence) after treatment with 100 μM 6-OHDA for 24 hours. Scale bar: 20 μm. In the magnifying figures (d Ctrl group and h 6-OHDA group), the scale bar is 10 μm. (**C–E**) The ROS levels (**C**) and intracellular Ca^2+^ levels (**D**) were measured by flow cytometry. The mRNA expression of ER stress genes CHOP, ATF3 were assessed by quantitative real-time PCR in the PC12 cells after treatment (**E**). For agitated Nur77, PC12 cells were incubated with 100 μM 6-OHDA or 5 μg/ml Csn-B alone, or in combination with both 6-OHDA and Csn-B, and control vehicle was used as control. For knocking-down Nur77, PC12 cells were transfected by empty vector or siRNA targeting Nur77 with or without incubation with 100 μM 6-OHDA. The bar chart shows the ROS levels (**C**) and intracellular Ca^2+^ levels (**D**) as the average intensity of the recorded fluorescence (NRFU) per cell (U/cell). (**F**) Western blots of *p*-AKT, AKT, *p*-PI3K, PI3K expressions in PC12 after treatment with 100 μM 6-OHDA or 5 μg/ml Csn-B alone, or in combination with both 6-OHDA and Csn-B, and control vehicle was used as control. The bar charts show the relative quantification of *p*-AKT/AKT and *p*-PI3K/PI3K. All the data are expressed as the relative ratios of the blank group, which was set to 1.0, and are expressed as the mean ± SEM of 5 independent experiments, **p < 0.01, ***p < 0.001.

Recent evidence suggest that Nur77-mediated apoptosis in tumor cells and T cells is associated with Nur77 nucleocytoplasmic translocation. Western blot and immunofluorescent staining with confocal microscopy was performed to determine the expression and localization of Nur77 in 6-OHDA-lesioned PC12 cells with or without Csn-B treatment. We extracted cytosolic fractions without mitochondria to detect Nur77 in endoplasm reticulum with/without 6-OHDA incubation. Nur77 increased to 11.5% in cytoplasm compared to the control group (n = 5 independent experiments, ***p < 0.001, Fig. 3A). In the 6-OHDA-treated group, PDI increased by 5.55 fold compared to the control (n = 5 independent experiments, ***p < 0.001, Fig. 3A). Moreover, our results showed that increased density of cytosolic Nur77 was co-localized with ER protein disulfide isomerase (PDI) in the 6-OHDA-treated group compared with control group (Fig. 3B).

We found that intracellular ROS levels increased by 1.67 fold in the 6-OHDA-treated group compared to the control, while Csn-B + 6-OHDA increased it by 2.22 fold (**p < 0.01, ***p < 0.001, n = 5 independent experiments, Fig. 3C). Consistently, we observed that the fluorescence intensity triangles of Csn-B with 6-OHDA-treated group shifted to the right compared to the one in 6-OHDA-treated group only, while the fluorescence intensity triangles of siNur77 with 6-OHDA treated group shifted to the left compared to the one in the siCtrl + 6-OHDA group (Fig. 3C). Similar results were also obtained from the alteration in intracellular Ca^2+^ with or without Csn-B treatment and with or without Nur77 expression (Fig. 3D). Moreover, we found that Nur77 regulated CHOP and ATF3 mRNA expression. CHOP mRNA expression in the group of Csn-B plus 6-OHDA increased by 9.0 fold, while 6-OHDA group increased by 5.7 fold compared to the control (6-OHDA vs. Csn-B + 6-OHDA, ***p < 0.001; Csn-B + 6-OHDA vs. Control group, ***p < 0.001, n = 5 independent experiments, Fig. 3E). ATF3 mRNA expression in the group of Csn-B with 6-OHDA increased by 9.5 fold, while 6-OHDA group increased by 8 fold above the control level (6-OHDA vs. Csn-B + 6-OHDA, **p < 0.001; Csn-B + 6-OHDA vs. Control group,***p < 0.001, n = 5 independent experiments, Fig. 3E), Strikingly, silencing Nur77 attenuated 6-OHDA-induced oxidative stress by downregulating CHOP and ATF3 mRNA levels. CHOP mRNA increased by 5.7 fold in the group of siCtrl plus 6-OHDA, while the groups of siNur77 with 6-OHDA only increased by 3.7 fold comparing with the siCtrl group. ATF3 mRNA levels in the siCtrl plus 6-OHDA group increased by 4.3 fold, while siNur77 plus 6-OHDA increased only by 2.7 fold (siNur77 + 6-OHDA vs. siCtrl; siNur77 + 6-OHDA vs. siCtrl + 6-OHDA, ***p < 0.001, n = 5 independent experiments, Fig. 3E). Consistently, the treatment of 6-OHDA with Csn-B downregulated the PI3K/AKT viability pathway, indicating that serious ER stress was activated to produce obvious apoptosis in the *in vitro* PD model (*p*-AKT: 50.4% of the control group in 6-OHDA group, 38.6% in the Csn-B + 6-OHDA group, n = 5 independent experiments, ***p < 0.001; *p*-PI3K: 52.4% of the control group in 6-OHDA-treated group, 38.6% in the Csn-B + 6-OHDA group, n = 5 independent experiments,***p < 0.001, Fig. 3F). These findings strongly demonstrated that Nur77 regulated the ER stress by increasing intracellular levels of ROS and Ca^2+^ and the expression of CHOP and ATF3 mRNA and downregulating PI3K/AKT signaling pathway.

### Nur77 induced mitochondrial impairment

**Figure 4:**
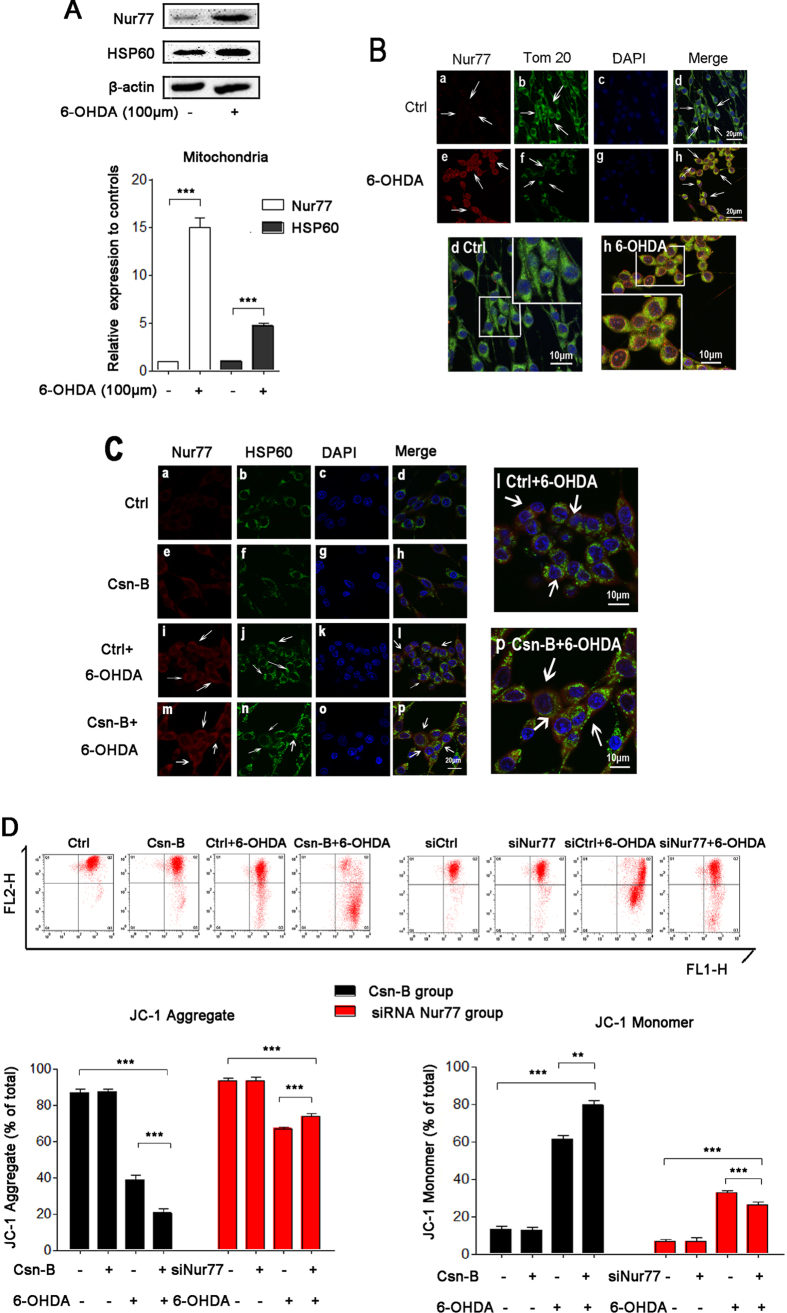
Nur77 induced PC12 cellular mitochondrial impairment. (**A**) Western blots of Nur77 and HSP60 in mitochondria after treatment with 100 μM 6-OHDA for 24 hours. The bar chart shows the relative quantification of Nur77, ***p < 0.001. (**B**) Immunofluorescent staining with confocal microscopy of Nur77 (red fluorescence) and Tom20 (green fluorescence) and DAPI (blue fluorescence) after treatment with 100 μM 6-OHDA for 24 hours. Scale bar: 20 μm. In the magnifying figures (d Ctrl group and h 6-OHDA group), the scale bar is 10 μm. (**C**) Immunofluorescent staining with confocal microscopy of Nur77 (red fluorescence) and HSP60 (green fluorescence) and DAPI (blue fluorescence) after treatment with 100 μM 6-OHDA or 5 μg/ml Csn-B alone, or in combination with both 6-OHDA and Csn-B, and control vehicle was used as control. Scale bar: 20 μm. The scale bar is 10 μm in the magnifying figures (d Ctrl group and h 6-OHDA group). (**D**) The mitochondrial membrane potential (∆Ψm) was measured by flow cytometry with JC-1 assay. For agitated Nur77, PC12 cells were incubated with 100 μM 6-OHDA or 5 μg/ml Csn-B alone, or in combination with both 6-OHDA and Csn-B, and control vehicle was used as control. For knocking-down Nur77, PC12 cells were transfected by empty vector or siRNA targeting Nur77 with or without incubation with 100 μM 6-OHDA. The bar chart on the left shows the JC-Aggregate rate (the unhealthy cells rate) of total cells. The bar chart on the right shows the JC-Monomer rate (the healthy cells rate) of the total cells. The results are expressed as the mean ± SEM of 5 independent experiments, **p < 0.01, ***p < 0.001.

We found that Nur77 increased by 15% in the mitochondria of 6-OHDA group compared to the control group in Nurr77 nucleocytoplasmic translocation (n = 5 independent experiments, ***p < 0.001, Fig. 4A). In the 6-OHDA group, HSP60 increased by 4.7 fold compared to the control (n = 5 independent experiments, ***p < 0.001, Fig. 4A). Our results showed that increased density of cytosolic Nur77 was co-localized with the mitochondrial out membrane protein Tom20 in the 6-OHDA-treated group (Fig. 4B) compared with the control group. We also found that Nur77 co-localized with Hsp60 both in the 6-OHDA group and Csn-B with 6-OHDA group, but the co-treatment with Csn-B and 6-OHDA increased the densities of Nur77 and HSP60 (Fig. 4C). This indicates that more Nur77 translocation from nuclear to mitochondria in 6-OHDA-lesioned PC12 cells is involved in mitochondrial impairment. To determine mitochondrial impairment, we used JC-1 staining to examine the mitochondrial membrane potential (ΔΨm). JC-1 aggregate rate represented the ratio of healthy cell with normal membrane potential to the total cells, while JC-1 Monomer rate represented the ratio of unhealthy cell with collapsed membrane potential to the total cells. Incubation with Csn-B showed little effect on the ΔΨm of PC12 cells. However, Csn-B significantly collapsed mitochondrial ΔΨm under 6-OHDA treatment, as JC-1 aggregate rate of Csn-B with 6-OHDA group decreased to 20.5% of the blank control level, while JC-1 aggregate rate in 6-OHDA alone group decreased to 38.9% of the control (n = 5 independent experiments, **p < 0.01, ***p < 0.001, Fig. 4D). In contrast, silencing Nur77 rescued the mitochondrial ΔΨm as the JC-1 aggregate rate was 73.7% in the siNur77 + 6-OHDA group and 67.0% in the siCtrl + 6-OHDA group (n = 5 independent experiments, **p < 0.01, ***p < 0.001, Fig. 4D).

### Nur77 induced autophagy through PINK1/Parkin/Beclin-1/p62

**Figure 5:**
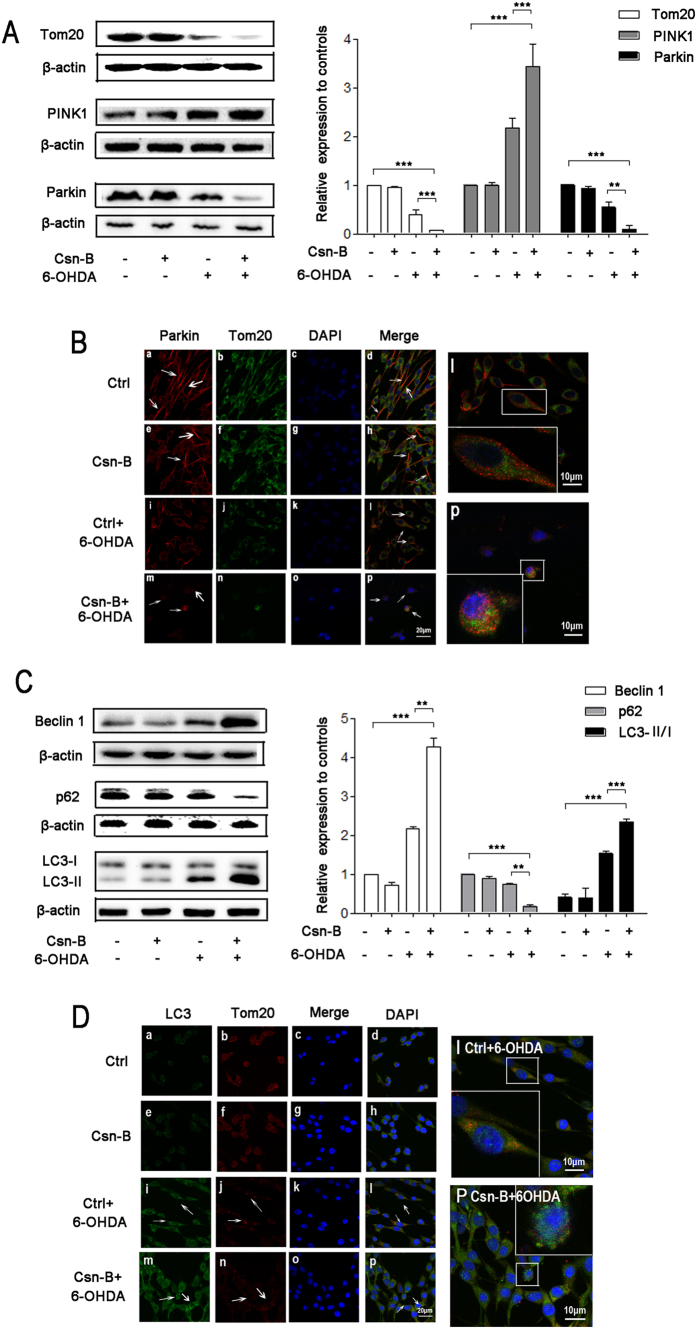
Nur77 induced autophagy through the mechanisms of PINK1/Parkin/Beclin-1/p62. (**A**) Western blots of Tom20, PINK1, Parkin expressions in PC12 after treatment with 100 μM 6-OHDA or 5 μg/ml Csn-B alone, or in combination with both 6-OHDA and Csn-B, and control vehicle was used as control. The bar chart shows the relative quantification of the protein levels compared with that of β-actin. The data are expressed as the relative ratios of the blank group, which was set to 1.0, and are expressed as the mean ± SEM of 5 independent experiments. **p < 0.01, ***p < 0.001. (**B**) Immunofluorescent staining with confocal microscopy of Parkin (red fluorescence) and Tom20 (green fluorescence) and DAPI (blue fluorescence) after treatment with 100 μM 6-OHDA or 5 μg/ml Csn-B alone, or in combination with both 6-OHDA and Csn-B, and control vehicle was used as control. Scale bar: 20 μm. In the magnifying figures (d Ctrl group and h 6-OHDA group), the scale bar is 10 μm. (**C**) Western blots of Beclin 1, p62, LC3 expressions in PC12 after treatment with 100 μM 6-OHDA or 5 μg/ml Csn-B alone, or in combination with both 6-OHDA and Csn-B, and control vehicle was used as control. The bar chart shows the relative quantification of the protein levels compared with that of β-actin. The data are expressed as the relative ratios of the blank group, which was set to 1.0, and are expressed as the mean ± SEM of 5 independent experiments. **p < 0.01, ***p < 0.001. (**D**) Immunofluorescent staining with confocal microscopy of LC3 (green fluorescence) and Tom20 (red fluorescence) and DAPI (blue fluorescence) after treatment with 100 μM 6-OHDA or 5 μg/ml Csn-B alone, or in combination with both 6-OHDA and Csn-B, and control vehicle was used as control. Scale bar: 20 μm. In the magnifying figures (d Ctrl group and h 6-OHDA group), the scale bar is 10 μm.

Our results showed that mitochondrial out membrane protein Tom 20 of Csn-B plus 6-OHDA group decreased to 7% of the control level, while Tom 20 of 6-OHDA alone group decreased to 38.8% of the control (n = 5 independent experiments, ***p < 0.001, Fig. 5A). Considering that Nur77 is involved in mitochondrial impairment, we proposed that Nur77 is also involved in mitochondrial autophagy.

Under the 6-OHDA condition, Csn-B increased PINK1 expression and decreased Parkin expression (PINK1: 2.2 fold higher in 6-OHDA group and 3.4 fold higher in the Csn-B + 6-OHDA group as compared to the control; Parkin levels as compared to the contrl: 54% in 6-OHDA group and 9% in the Csn-B + 6-OHDA group, n = 5 independent experiments, **p < 0.01, ***p < 0.001, Fig. 5C). In confocal microscopy studies, we observed that Parkin distributed mainly in the cytoplasm but not in the mitochondria. Most of the Parkin did not co-locate with Tom20 in normal condition. However, 6-OHDA induced Parkin accumulation on the mitochondria membrane. The co-location of Tom20 and Parkin in the 6-OHDA group and Csn-B with6-OHDA group, and Parkin density in Csn-B plus 6-OHDA group decreased sharply (Fig. 5B). This indicated that damaged mitochondria were removed via mitochondrial autophagy. In term of the autophagy markers, 6-OHDA treatment slightly increased the Beclin 1 and ratio of LC3 II to LC3 I (Beclin 1: 1.2 fold of the control group; the ratio of LC3 II to LC3 I 1.5 vs. 0.4 in the control group; n = 5 independent experiments, Fig. 5C). In contrast, 6-OHDA decreased p62 to 73.5% of the control level (n = 5 independent experiments, **p < 0.01, ***p < 0.001, Fig. 5C). However, Csn-B promoted the autophagy process as the Western blot results showed that Beclin 1 and the ratio of LC3 II to LC3 I significantly increased by 1.8 fold and by 2.3 fold, respectively, and p62 decreased to17.5% of the control in the Csn-B + 6-OHDA group compared with 6-OHDA group (n = 5, independent experiments, **p < 0.01, ***p < 0.001, Fig. 5C).

We also found that LC3 co-location with Tom20 after treatment with 6-OHDA (Fig. 5D) and a decreased density of Tom 20 and increased density of LC3 under co-treatment of 6-OHDA and Csn-B (Fig. 5D). Interestingly, in the Can-B with 6-OHDA group, LC3 and Tom20 were fragmented in some cells, indicating the degradation of mitochondria (Fig. 5D(p)).

## Discussion

We investigated the roles of Nur77 in PC12 cell injury induced by 6-OHDA and made three novel findings in this study: (1) Nur77 knockdown attenuates 6-OHDA-induced PC12 cell injury, while its activation exacerbates cellular degeneration; (2) Nur77 displays a devastating effects on 6-OHDA-lesioned PC12 cells partially through exacerbating mitochondrial impairment and ER stress; and (3) Nur77 enhances autophagy. To our knowledge, this study is the first to demonstrate that ER stress inducing caspase-dependent apoptosis and autophagic cellular death in 6-OHDA-lesioned PC12 cells is mediated by Nur77 signaling. Even though they are not considered neurons, PC12 cells can easily differentiate into neuron-like cells and share properties similar to neurons, which make PC12 cells useful as a model system for neuronal differentiation^21^. Though our results are not from midbrain dopaminergic neurons, our study using PC12 cells displaying an unspecific catecholaminergic phenotype, still provides a crucial clue that Nur77 may be an important modulator in mediating mitochondrial impairment and cellular death in the pathogenesis of PD.

Several lines of evidence suggest that Nur77 plays important roles in DA neuronal death, differentiation, biochemical activity, dopamine turnover, dopamine-related neuroadaptation processes^22^,^23^,^24^,^25^. Specifically, under the nuclear export signals induced by nerve growth factor (NGF)^25^, Nur77 modulates retinoid signaling through heterodimerization with retinoid-X receptor^24^. One *in vivo* study by St-Hilaire *et al*.,^25^ showed that in Nur77^−/−^ mice, Nur77 proved to set the threshold level for L-DOPA-induced rotational behavior. L-DOPA-induced rotational response was exacerbated in Nur77^−/−^ mice, compared to Nur77^+/+^ ones, while the up-regulation of encephalin and neurotensin by lesioning the nigrostriatal pathway was significantly reduced in Nur77^−/−^ mice. In terms of behavioral and molecular adaptations to dopamine denervation and repeated L-DOPA treatment, it does seem to be worsening in Nur77^−/−^ mice. Therefore, Nur77 may play different roles in different conditions. The total levels of Nur77 increased in a dose-dependent manner under 6-OHDA lesion (Fig. S1). Our findings (Figs 3A and 4A) showed that under 6-OHDA treatment, Nur77 accumulated in cytosol and mitochondria that is consistent with Martinoli’ study^26^ indicating that 6-OHDA induced the translocation of Nur77. This implies that in 6-OHDA induced cellular death, Nur77 is concurrently contributing to DA neuronal death as a detrimental factor. To investigate the mechanisms of Nur77, we further activated or blocked Nur77 function using its agonist and knockdown respectively in the *in vitro* model. These findings (Figs 1D–F and 2A) show that Nur77 was positively parallel with the 6-OHDA-mediated injury and aggregated the neurodegeneration in PC12, and further confirming our conclusion that Nur77 may induce deleterious effects on this *in vitro* model.

An important observation in this study was that in 6-OHDA lesion, Nur77 translocated from nuclear to mitochondria and endoplasm reticulum. Given this translocation to both mitochondria and endoplasm reticulum, we propose that this Nur77 translocation from the nucleus into the cytosol may initiate the cellular death. Deficiencies in mitochondrial dynamics and ER stress under the conditions of Ca^2+^ homeostasis, oxidative stress and apoptosis not only result in cellular abnormal energy metabolism, but also accelerate neurodegeneration^26^,^27^. Indeed, Merkwirth *et al*., indicated that mitochondrial defects including the perinuclear clustering of mitochondria in hippocampal neurons, mitochondrial fusion and mitochondrial ultrastructure disruption lead to extensive neuro- degeneration associated with behavioral impairments and cognitive deficiencies in mice^28^.

Previous evidence has shown that Nur77 translocation to cytoplasm is necessary for ER-induced stress^29^,^30^. It has been documented that severe ER stress can induce neuronal death and exacerbate neurodegeneration via different pathways including autophagic induction, cytotoxicity, upregulation of both CHOP and p53, ER mitochondria calcium cycle and apoptosis^3^,^31^,^32^,^33^,^34^,^35^,^36^,^37^. CHOP, a transcription factor, is reported as an ER-stress induced modulator in cellular death and the cascade CHOP-ATF3 is involved in ER-stress^38^. The observation that under oxidative stress the levels of CHOP-ATF3 and *p*-AKT/*p*-PI3K were profoundly increased and decreased respectively implies that serious ER stress was activated to produce obvious apoptosis in the *in vitro* model (Fig. 3E,F). In addition, the increased CHOP together with decreased Bcl 2 following Nur77 activation under oxidative stress suggestes that Nur77 may regulate ER stress and influence the cellular survival, which is consistent with one previous study showing CHOP down-regulated Bcl2 and making cells sensitive to ER stress^39^.

Interestingly, the present study showed that CHOP-ATF3 was upregulated or downregulated in 6-OHDA-lesioned PC12 in response to Nur77 activation or knockdown, respectively. It also showed that p-AKT/p-PI3K downregulation following Nur77 activation with 6-OHDA lesion only. This finding further indicates that the translocation of Nur77 from the nuclear may directly exacerbate ER stress and trigger cellular death via CHOP-ATF3 and AKT-PI3K signalling pathways. It is noteworhty that our results are different from thoses of previous studies, showing that mild ER stress inhibits neuronal death by promoting autophagy or mitophagy^17^,^40^. The discrepant results may be due to the fact that 6-OHDA (100 μM) in our study induced severe ER stress that stimulates and aggravates both apoptosis and cellular death. This is while ER stress was relatively mild in previous studies and therefore probably induced autophagy/mitophagy mediated neuroprotection.

In addition to ER stress, mitochondrial impairment was also observed under oxidative stress and aggregated following Nur77 activation, as indicated by HSP60 and Cyt C. We hypothesize that the Nur77 translocation initiates the neuronal death probably through disruption of mitochondrial integrity, excessive mitochondria clearance and further induction of autophagy, subsequently leading to an irreversible cellular death^14^,^41^. The increased intracellular calcium (Fig. 3D) may influence the sensitivity of mitochondria to oxidative stress, and further aggravates the 6-OHDA-mediated mitochondrial impairment, which may lead to an irreversible cellular death. Previous studies suggest that the subcellular location of NR4A is functionally important and cytoplasmic NR4A acts on the mitochondria and interacts with Bc12 to promote apoptosis^42^,^43^. For example, Lin *et al*.^44^ and Thompson *et al*.^43^, showed that translocation of Nur77 from nuclear to mitochondria promoted its interaction with Bcl2 and switched Bcl2 activity from an anti-apoptotic factor into a pro-apoptotic molecule in cancer cells and T cells. In this study, we found that 6-OHDA-induced translocation of Nur77 into the cytosol is enhanced following Nur77 activation and this enhanced translocation of Nur77 is parallel with the downregulation of Bcl2, upregulation of caspase 3, and cellular apoptosis. Therefore, it seems that Nur77 does not only switch Bcl2 activity from an anti-apoptotic factor into a pro-apoptotic molecule, but also reduces the level of Bcl2, leading cell injury. These findings demonstrate that Nur77 positively modulates the mitochondrial impairment and promotes apoptosis via regulating Bcl 2 and caspase 3 under oxidative stress. The fact that the 6-OHDA-induced apoptosis was attenuated after Nur77 knockdown further supports the critical role of Nur77 in cellular death. In addition, we identified the mitochondrial function using JC-1 assay to detect the mitochondrial membrane potential and evaluate its function. Our results showed that the 6-OHDA-induced a sharp loss of ΔΨm and increased JC-1. This was attenuated and aggregated by Nur77 activation and knockdown respectively. Subsequently this verified that Nur77 may be directly involved in the modulation of mitochondrial membrane function. Based on the above observations, the present mechanisms identified in PC12 cells support a role of Nur77 in DA cell loss. However, these findings need to be further validated in other *in vitro* and *in vivo* PD models.

As the markers of mitochondria functions, PINK1 and Parkin (encoded by the *PARK2* gene) are the key proteins associated with autophagy in the neuro-pathogenesis of PD. They physically associate and functionally label damaged mitochondria for selective degradation via autophagy^45^,^46^,^47^,^48^. Choubey *et al*., showed that Beclin 1, the autophagy protein, is involved in PARK2 translocation to mitochondria and regulates PINK1 overexpression-induced PARK2 translocation^49^. Wong and Holzbaur indicated that through the LC3-interacting region, another autophagy-related protein, autophagosome assembly around mitochondria was induced after ubiquitinating mitochondria downstream of *PARK2* which interacted with OPTN (optineurin)^50^.

To further determine whether Nur77 affects 6-OHDA-lesioned PC12 cells via regulating autophagy and mitochondrial functions and influencing PINK1 and Parkin, we activated Nur77 and then examined the association of autophagy and mitochondrial functions together with PINK1/Parkin levels. Beclin-1 acts as a promoter in autophagy, while p62 is the substance of autophagy and usually behaves as a cargo receptor for ubiquitinated proteins to play a role in selectively delivering various proteins to the autophagosome. The increased Beclin-1 and decreased p62 imply an increased autophagic induction. Our results (Fig. 5A,B) strongly verified that Nur77 has a harmful influence on mitochondrial function. Interestingly, the expressions of Betclin 1 and LC3 are similar to that of PINK1 but contrary to Parkin with Nur77 activation under oxidative stress. On the other hand, the alteration in p62 is different from those of both Beclin-1 and LC3. Our observation on the colocation of Parkin/Tom20 and LC3/Tom20 is consistent with the viewpoint that Parkin binds to depolarized mitochondria and induced autophagy of mitochondria^51^. Both Parkin and PINK1 were involved in cellular response to Nur77 agonist under 6-OHDA condition for up-regulation of PINK1 and down-regulation of Parkin after Nur77 agonist treatment. We propose that PINK1 may act as an upstream factor for Parkin to initiate the autophagic degradation of damaged mitiochondria and is essential for recruiting Parkin from the cytoplasm to depolarized mitochondria to some extent, responding to Nur77^52^,^53^. PINK1 on the mitochondria is regulated by voltage-dependent proteolysis to maintain low levels of PINK1 for rapidly and constitutively degradation under steady-state conditions, while the loss in mitochondrial membrane potential stabilizes PINK1 mitochondrial accumulation^51^,^53^. This may be the one of the potential mechanisms of neurotoxicity linked to increase in PINK1. However, there is little evidence showing that the downregulation of PINK1 is directly linked to PD progression. Taken together, this finding strongly suggests that Nur77 may directly regulate cellular autophagy by modulating mitochondrial functions. Mitochondrial potential collapse oppositely influences PINK1 and Parkin. These findings further confirmed that Nur77 may be used as the autophagic modulator by influencing mitochondrial target in the *in vitro* PD model.

## Conclusions

**Figure 6:**
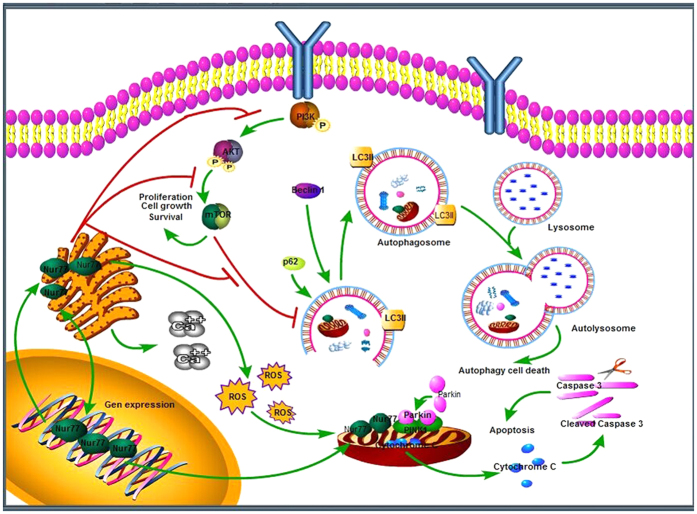
Schematic illustration of Nur77 signaling mechanisms in 6-OHDA lesioned PC12 cells. All of cropped gels/blots used in the main figures (Figs 1, 2, 3, 4 and 5) have been run under the same experimental conditions. The rectangles encompassing gels/blots (Figs 1A–C,F, 2B, 3A,F, 4A and 5A,C) are cropping lines.

In summary, we have demonstrated an increase in the cytosolic Nur77 and translocation from the nucleus together with mitochondrial impairment as indicated by the upregulation of cytosolic HSP60/Cyt C and increased JC-1 as well as downregulation of Tom20 and sharply loss of ΔΨm following 6-OHDA lesion, while Nur77 activation/knockdown aggravates/attenuates such pathophysiological changes respectively. In addition, Nur77 exacerbates PC12 cellular death by upregulating Beclin-1/LC3, PINK1, CHOP and ATF3 as well as downregulating Parkin in the *in vitro* model, indicating enhanced autophagic induction and ER stress. Moreover, Nur77 exacerbates Ca^2+^ homeostasis, ROS and LDH in the *in vitro* model. We hypothesize that Nur77 may not only regulate mitochondrial dysfunction, but also modulate endoplasm reticulum stress in the development of neurodegenerative diseases like PD. Importantly, our proposed mechanisms for Nur77 in inducing autophagy and modulating mitochondrial dysfunctions and ER stress in PD pathogenesis increase our understanding of the roles of Nur77 in the disease (Fig. 6). This study provides a clue for the development of an alternative approach to the treatment of neurodegenerative diseases like PD by modulating Nur77-mediated mitochondrial impairment and ER stress as well as Nur77-inducted enhanced autophagy. A better understanding of the roles and relationships between Nur77, mitochondrial dysfunction, ER stress and autophagy may open new perspectives for the treatment of neurodegenerative disorders such as PD by targeting Nur77 signaling.

## Materials and Methods

### Cell culture and treatment

PC12 cells were prepared as described in our previous study and other studies^2^,^54^. Briefly, PC12 cells were routinely maintained in Dulbecco’s modified Eagle’s medium (DMEM) supplemented with 10% fetal bovine serum, 5% horse serum, 100 U/ml benzyl penicillin, and 100 g/ml streptomycin (Gibco Life Technologies, Rockville, MD, USA). For all experiments, the cells were seeded in 96-well plates or 6-well plates at a density of 1.0 × 10^5^ cells/ml for 24 h. Then, four experimental groups were treated with either media (DMEM), Csn-B (5 μg/ml) only, 6-OHDA (100 μM), or 6-OHDA (100 μM) + Csn-B (5 ug/ml).

### Construction of the Nur77 siRNA Sequence and Its Transfection into PC12 Cells

Three Nur77 siRNA oligonucleotides were purchased and identified using the primers S1 (5′-CCGCUUUGGAAAGGAAGAUTT-3′), S2 (5′-CGGCU UCCUUUAAGUUUGATT-3′) and S3 (5′-CCUGUAUCCAAGCUCAAUATT-3′). The negative control sequence (Sn, 5′-UUCUCCGAACGUGUCACGUTT-3′) was formulated and synthesized. Transfection was performed using Lipofectamine RNAiMAX Transfection Reagent (Life Technologies Corp, Carlsbad, CA, USA) according to the manufacturer’s instruction. The transduction efficiency was evaluated by real-time-PCR (Nur77, forward primer, 5′-CGACCCCCTGACCCCTGAGTT-3′, reverse primer, 5′-GCCCTCAAGGT GTTGGAGAAGT-3′). Only the most effective siRNA (S1) was used in the subsequent studies. The experimental groups were divided into four groups: negative control transfected with empty vector (siCtrl), siRNA targeting Nur77 (siNur77), negative control transfected with empty vector plus 6-OHDA (siCtrl + 6-OHDA) and siRNA targeting Nur77 plus 6-OHDA (siNur77 + 6-OHDA).

### MTS Assay, LDH and Apoptotic Cell Measurement

Cell viability was measured using CellTiter96 Aqueous Assay (Promega, Madison, WI, Cat: G3582). Briefly, after 6-OHDA incubation for 24 hrs, 20 ul/well of CellTiter96 Aqueous was added and control wells without cells contained culture medium and CellTiter96 Aqueous. One solution was set to correct background 490 nm absorbance, and the absorbance at 490 nm was recorded using a microplate reader (BioTek Instruments Inc., Missouri, USA). Survival of control group was defined as 100%, and other groups were expressed as percentage of the control group. To further investigate the apoptosis, PC12 cells were seeded at a density of 1 × 10^5 ^cells/well in 6-well plates. After incubation with 6-OHDA for 24 hrs, PC12 cell apoptosis was evaluated by flow cytometry (Bender MedSystems, Burlingame, CA, USA).

The cytotoxicity was evaluated by measuring the LDH leakage into the culture medium using Cytoscan-LDH cytotoxicity assay kit (G-Biosciences Inc., St Louis, MO, USA). After 6-OHDA incubation for 24 hrs, 50 μL supernatant of each wells were transferred to another fresh 96-well plate and mixed with 50 μL reconstituted Substrate Mix, and then these mixtures were incubated at 37 °C and protected from light for 30 mins. Lastly, 50 μL Stop Solution was added to each well to terminate the reaction. The absorbance of the solution was recorded by Microplate Reader with the absorbance wavelength at 490 nm.

### Measurement of intracellular ROS and calcium concentrations

The level of intracellular ROS was measured using a ROS kit. The cells were collected and rinsed twice with PBS then they were incubated with 10 μM of the cell permeable 2′,7′-dichlorofluorescin diacetate (DCFH-DA) for 20 minutes at 37 °C. After that, the cells were rinsed with medium without FBS three times, and then their fluorescence was detected using a flow cytometer. The results were presented as an average of the intensity of the recorded fluorescence (NRFU) (U/cell).

The intracellular Ca^2+^ levels in PC12 cells were measured using the fluorescence Ca^2+^ indicator Fluo-3 AM. The cells were collected, rinsed twice with PBS, and stained with 5 μg/ml of Fura-3AM for 60 minutes. After staining, the cells were rinsed twice with PBS and incubated for 30 minutes at 37 °C before fluorescence-intensity detection by a flow cytometer. Calcium levels in cells were expressed as NRFU (U/cell).

### Measurement of mitochondrial membrane potential (∆Ψm)

∆Ψm of PC12 cells was determined by flow cytometry according to the manufacturer’s protocol using JC-1 mitochondrial membrane potential assay kit (Cayman Chemical Co., Ann Arbor, MI, USA). Briefly, cells in 6 well plates were seeded at the density of 5 × 10^5 ^cells/ml in a CO2 incubator before being treated with 6-OHDA. After 24 hrs, cells from each well were harvested into a plastic tube and rinsed with PBS twice. 50 μL of the JC-1 Staining Solution per 500 μL of culture medium was added to each tube and then incubated in a CO_2_ incubator at 37 °C for 25 minutes. Healthy cells with functional mitochondria contained red JC-1 J-aggregates and were detectable in the FL2 channel. Unhealthy cells with collapsed mitochondria contained mainly green JC-1 monomer and were detectable in the FL1 channel.

### Preparation of cytosolic and mitochondrial fractions and Western blotting

To obtain separate mitochondrial, cytosolic and nuclear fractions, cells were suspended in 2 ml MS buffer (210 mM mannitol, 70 mM sucrose, 5 mM Tris-HCl, pH 7.5 and 1 mM EDTA) containing 1% protease inhibitor cocktail, and then homogenized using a Dounce homogenizer. The homogenate was spun at 1300 g for 10 minutes at 4 °C to pellet nuclei and unbroken cells. The pellet was washed with ice-cold PBS and re-suspended in MS buffer. The nuclei were purified by a second round of centrifugation at 1300 g. The supernatant containing the heavy-membrane (HM) fraction enriched with mitochondria was subjected to centrifugation at 17,000 g for 30 minutes at 4 °C. After centrifugation, the supernatant was collected as the cytosolic fraction, and the pellet was further re-suspended in 3 ml MS buffer and layered on a sucrose gradient: 2 ml of 1 M sucrose buffer and 5 ml of 1.5 M sucrose buffer (10 mM Tris-HCl at pH 7.5 and 1 mM EDTA), which was centrifuged at 60,000 g for 30 minutes at 4 °C. Gradient-purified mitochondria was collected at the interface of the sucrose gradients and dissolved in the dilution buffer (5 mM Tris-HCl, pH 7.5 and 1 mM EDTA). Cell lysates as the whole protein were made using RIPA buffer (Thermo Scientific) containing 25 mM Tris•HCl pH 7.6, 150 mM NaCl, 1% NP-40, 1% sodium deoxycholate, 0.1% SDS and proteinase inhibitor cocktail containing 2 mM PMSF, 20 μg/ml aprotinin, 10 μg/ml leupeptin. The concentration of the protein was determined by bicinchoninic acid (BCA) assay (Pierce, Inc., Rockford, IL, USA) and protein was separated by SDS-PAGE and electrically transferred to a polyvinylidene fluoride (PVDF) membranes (Amersham Bioscience, Ltd., Buckinghamshire, UK). The membranes were blocked in 5% skim milk, 0.05% Tween 20, and Tris-buffered saline (TBS) for 1 h. The PVDF membranes were incubated in the following primary antibodies overnight at 4 °C: rabbit anti-Nurr1 (Sigma; 1:500, **Cat: N6413**), rabbit anti-Nur77 (Proteintech; 1:500, **Cat: 12235-1-AP**), rabbit anti-tyrosine hydroxylase (TH) (Santa Cruz Biotechnology; 1:500, **Cat: sc-14007**), rabbit anti-dopamine transporter (DAT) (Santa; 1:500, **Cat: sc-14002**), rabbit anti-phosphatidylinositol 3 kinase (PI3K)/PI3K (Cell Signaling; 1:500, **Cat: 4228/4249**), rabbit anti- p-AKT/AKT (Cell Signaling; 1:500**, Cat: 4060/9272**), mouse anti-Cytochrome C (Abcam; 1:1000, **Cat: ab13575**), rabbit anti-cleaved caspase 3(Cell Signaling;1:1000, **Cat: 9661**), rabbit anti-bcl2 (abcam;1:200, **Cat: ab7973**), rabbit anti-beclin1(Cell Signalin;1:100, **Cat: 3738**), rabbit anti-LC3(Cell Signaling;1:1000, **Cat: 4108**), rabbit anti-β-actin (Cell Signaling; 1:1000, **Cat: 4967**). The images were analyzed using NIH Image J software.

### Immunofluorescent staining

Cells were plated on confocal Petri dishes in serum-containing media for 24 hrs before treatments. After different treatments, the dishes were washed in phosphate buffer saline (PBS), and then fixation of the cells was conducted with 4% paraformaldehyde in PBS for 15 min at room temperature. Then the cells were rinsed with PBS and incubated with PBS-T (0.3% Triton X-100 in PBS) for permeabilization for 15 min. After three more washes with PBS, the cells were incubated with blocking buffer [PBS, 3% bovine serum albumin (BSA)] for 60 min at room temperature. The following primary antibodies were used: goat anti-Nur77 (Sigma; 1:200, **Cat: sc-7013**), mouse anti-Cyt C (Abcam; 1:250,**Cat: ab13575**), and rabbit anti-HSP60 (Cell Signaling; 1:800, Cat:12165), rabbit anti-PDI (Cell Signaling; 1:500, **Cat: 2446**) rabbit anti-Parkin (Abcam; 1:200, **Cat: ab15954**), rabbit anti-cleaved caspase 3(Cell Signaling;1:200, **Cat: 9661**), goat anti-MAP LC3 (Santa; 1:100, **Cat: sc-54237**), mouse anti-Tom20 (Santa; 1:100, **Cat: 17764**). The specimens were incubated with the primary antibodies in PBS containing 3% BSA overnight at 4 °C. For immunofluorescence, on the following day, after additional rinsing with PBS, the samples were incubated with fluorescent-labeled secondary antibodies (Alexa 488-, Alexa 568-, Alexa 555- or Alexa 647-labeled IgG; Invitrogen) in PBS containing 3% BSA for 60 min at room temperature. After rinsing with PBS, 4′,6-Diamidino-2-phenylindole dihydrochloride (DAPI) (300 nmol/L, Invitrogen, Carlsbad, CA, USA) was used for counterstaining for 3–5 min. The confocal analysis was performed using a Zeiss LSM 710/Meta Station (Carl Zeiss) equipped with a digital camera (Himamatsu, Japan) and operated by QED imaging software.

### RT-PCR Determination

Cells were harvested and total RNA was extracted using TRIzol^R^ Reagent (Invitrogen, Grand Island, NY, Cat: 15596-018) and reversely transcribed into cDNA by Transcriptor First Strand cDNA Synthsis Kit (Roche, Germany). Real Time-PCR was carried out using LightCycler 480SYBR Green I Master (Roche) according to the manufacturer’s instruction. cDNA was amplified using CHOP and ATF3 as primers. Levels of β-action were used as normalization controls. The primers used included rat-specific β-action: forward primer, 5′-TTGCTGACAGGATGCA GAAG-3′; reverse primer, 5′-CAGTGAGGC CAGGATAGAGC-3′; GADD153/CHOP: forward primer, 5′-AGCTGA GTCTCTGCCTTTCG-3′; reverse primer, 5′-CGTTTCCTGGGGATG AGATA-3′, ATF3: forward primer, 5′-CTCCTGGGTCACTGGTGTTT-3′; reverse primer, 5′-ATGGCAAAGGT GCTTGTTCT-3′.

### Statistical Analysis

The data are expressed as the mean ± standard error of the mean (SEM). The data were analyzed using a one-way analysis of variance (ANOVA) followed by Bonferroni’s comparison post hoc analysis (SPSS 15.0 program, Chicago, IL). Differences with *p* values of less than 0.05 were regarded as statistically significant.

## Additional Information

**How to cite this article**: Gao, H. *et al*. Nur77 exacerbates PC12 cellular injury *in vitro* by aggravating mitochondrial impairment and endoplasmic reticulum stress. *Sci. Rep.* **6**, 34403; doi: 10.1038/srep34403 (2016).

## Supplementary Material

Supplementary Information
