# Supplementary material for: Nur77 exacerbates PC12 cellular injury in vitro by aggravating mitochondrial impairment and endoplasmic reticulum stress
Source: Sci Rep. 2016 Sep 29;6:34403. doi: 10.1038/srep34403 (PMC5041156; doi:10.1038/srep34403)
Supplement: Supplementary Information [file srep34403-s1.docx]

# Nur77 exacerbates PC12 cellular injury *in vitro* by aggravating mitochondrial impairment and endoplasmic reticulum stress

Huimin Gao ^1†^, Zhaoyu Chen ^1†^, Yongmei Fu ^2†^, Xiaoyan Yang ^2†^, Ruihui Weng ^1^, Rui Wang ^1^, Jianjun Lu ^6^, Mengqiu Pan^6^, Kunlin Jin ^3^, Chris McElroy ^3^, Beisha Tang ^5^, Ying Xia ^4^*, Qing Wang ^1^*


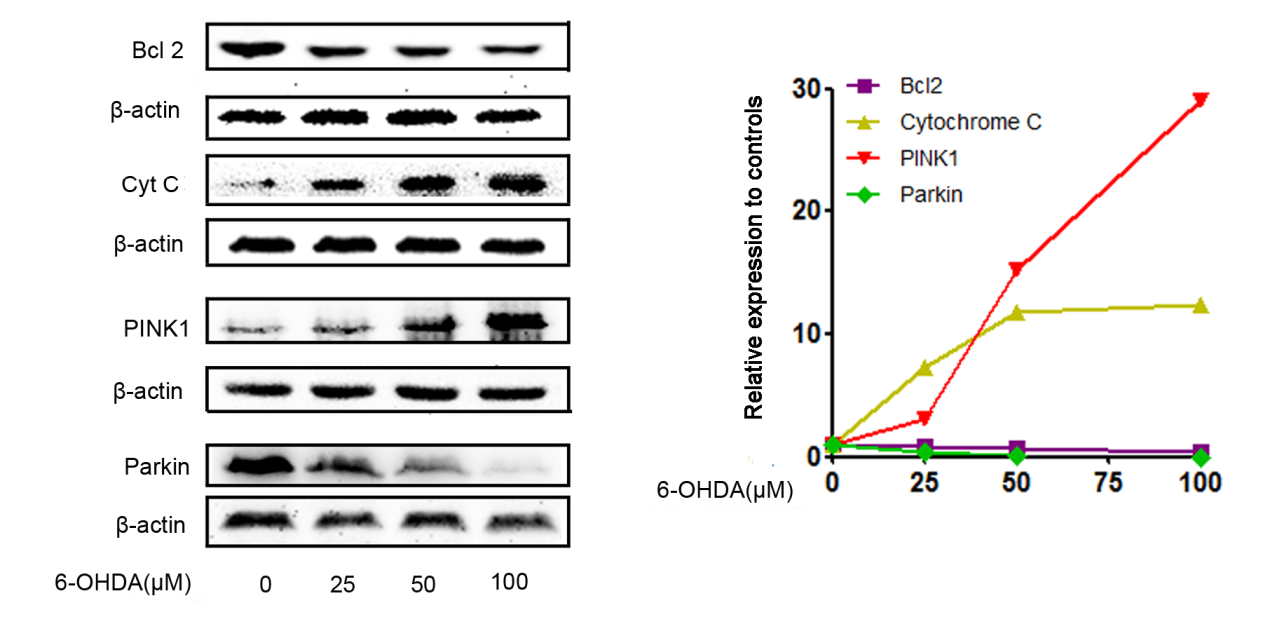


**Supplementary Figure 1**

The Western blots of Bcl2, Cytochrome C, PINK1, Parkin expression in PC12 under different concentration of 6-OHDA (0, 25, 50, 100 µM) for 24 hours. The line chart shows the relative quantification of the specific protein levels compared with that of β-actin. The data are expressed as the relative ratios of the blank group, which was set to 1.0, and are expressed as the mean of 5 independent experiments
